# Effects of propolis supplementation on irritable bowel syndrome with constipation (IBS‐C) and mixed (IBS‐M) stool pattern: A randomized, double‐blind clinical trial

**DOI:** 10.1002/fsn3.2806

**Published:** 2022-04-20

**Authors:** Mahsa Miryan, Davood Soleimani, Pejman Alavinejad, Mohammadreza Abbaspour, Alireza Ostadrahimi

**Affiliations:** ^1^ 48432 Student Research Committee Tabriz University of Medical Sciences Tabriz Iran; ^2^ 48432 Nutrition Research Center Department of Clinical Nutrition School of Nutrition and Food Sciences Tabriz University of Medical Sciences Tabriz Iran; ^3^ 48464 Nutritional Sciences Department School of Nutritional Sciences and Food Technology Kermanshah University of Medical Sciences Kermanshah Iran; ^4^ 48464 Research Center of Oils and Fats Kermanshah University of Medical Sciences Kermanshah Iran; ^5^ Hepatology Department School of Medicine Jundishapur University of Medical Sciences Ahvaz Iran; ^6^ 37552 Targeted Drug Delivery Research Center Pharmaceutical Technology Institute Mashhad University of Medical Sciences Mashhad Iran

**Keywords:** gastrointestinal diseases, IBS, irritable bowel syndrome, propolis, Rome IV criteria

## Abstract

**Background:**

Recent evidence indicates that propolis can modulate gastrointestinal (GI) function. This trial aims to assess the efficacy of propolis supplementation on the severity of irritable bowel syndrome (IBS) symptoms.

**Methods:**

This clinical trial was conducted on 56 subjects with IBS diagnosed by Rome IV criteria. Eligible subjects were randomly assigned to receive either 900 mg/day of propolis or matching placebo tablets for 6 weeks. The IBS symptom severity scale (IBS‐SSS) was used to evaluate IBS severity in five clinically applicable items.

**Results:**

After adjusting anxiety scores, a significant reduction was observed in the overall score of IBS symptoms (−98.27 ± 105.44), the severity of abdominal pain (−24.75 ± 28.66), and the frequency of abdominal pain (−2.24 ± 3.51) with propolis treatment as compared to placebo (*p*‐value < .05). Patients in the propolis group were 6.22 times more likely to experience improvement in IBS symptoms than those in the placebo group (95% CI: 1.14–33.9; *p*‐value: .035). There was no significant change in anthropometric measurements and dietary intakes in both groups (*p*‐value > .05).

**Conclusions:**

Our results showed that propolis supplementation might have a beneficial effect on constipation subtype of IBS (IBS‐C) and mixed subtype of IBS (IBS‐M) severity by reducing the severity and frequency of abdominal pain in patients with irritable bowel syndrome (IBS).

## Graphical abstract

Our results showed that propolis supplementation might have a beneficial effect on constipation subtype of IBS (IBS‐C) and mixed subtype of IBS (IBS‐M) severity by reducing the severity and frequency of abdominal pain in patients with IBS.

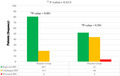

## 1 INTRODUCTION

Irritable bowel syndrome (IBS) is a common functional gastrointestinal (GI) disease that is manifested by recurrent abdominal pain and altered bowel function (Rodiño‐Janeiro et al., 2018). No specific markers or laboratory parameters are available to diagnose the disease. Recently in clinical practice, the Rome IV criteria have been proposed as the latest diagnostic tool for IBS, based on the GI symptoms (Bai et al., 2017). IBS is estimated to affect women approximately three times more than men, with an overall prevalence of 10% (Collins et al., 2001; Ishihara et al., 2019).

The pathophysiology of IBS is not well known, but several factors have been attributed to an individual's susceptibility to IBS including the alterations in gut microbiota, gut–brain axis, gut motility or/and permeability, and intestinal immune system function; GI microscopic inflammation; psychological stress; chronic infections; specific nutrients and foods; and genetic factors (Saha, 2014). Recent investigations also reveal the role of inflammatory and oxidative stress factors in increasing nervous system sensitivity and perception of abdominal pain in IBS subjects (Collins et al., 2001). Various strategies are recommended to improve or even treat IBS symptoms, but often with little success so far (Xu et al., 2019). The current management strategy of IBS is based on the prohibition of consuming gas‐producing foods including fermentable oligosaccharides, disaccharides, monosaccharides, and polyols (FODMAPs). FODMAP foods contain high amounts of carbohydrates that the body cannot absorb properly (e.g., apples, cherries, pears, peaches, artichokes, asparagus, onions, garlic, beans, lentils, cashews, pistachios, wheat, rye, dairy‐based milk, yogurt, and ice cream). The low FODMAP regimen is not a healthy diet for life; recent studies have shown that following this diet in the long term may have a negative effect on patients’ health. The biggest downside to the FODMAP diet has been the perception that it decays the microbes. Controlled studies have shown that reducing the use of FODMAP does not affect bacterial diversity, but reduces the total abundance of bacteria, and increasing the use of FODMAP increases health‐promoting bacteria. This version of the diet is commonly used in practice by experienced FODMAP‐trained nutritionists but is not clearly explained in studies. Accurate orientation and evaluation of the response or nonresponse reduce the risk of an excessive and less restrictive diet (Halmos & Gibson, 2019).

Emerging evidence has shown an important role of the modulating GI immune system and gut microbiota using prebiotic and/or probiotic supplements in ameliorating the symptoms of IBS, which has been beneficial for many patients (Moayyedi et al., 2010). Dietary polyphenols and their secondary metabolites also have a crucial role in maintaining the balance of the GI microbiome by altering bacterial metabolites that can raise mucin gene expression, resulting in an increase in the thickness of the GI mucosal layer and also reduction in GI inflammation (Suzuki & Hara, 2011).

Propolis, a popular traditional medicine, is a resinous hive product collected by honeybees from varied petals and plant buds sources (Nina et al., 2015). With the advent of new methods such as high‐performance liquid chromatography (HPLC), more than 300 types of phytochemicals have been identified in this hive product, mainly from the family of polyphenols. They are secondary plant metabolites with well‐known antioxidant properties (Cornara et al., 2017). Recent studies have shown that propolis, due to the unique diversity of its components (especially polyphenols), not only has antioxidant effects but can also modulate the inflammatory pathways, immune system function, gut microbiota, and GI permeability (Jalali et al., 2020; Wang et al., 2016; Xue et al., 2019). Considering the wide range of probable causes and symptoms in IBS patients that propolis may modify, we aimed to assess the efficacy of propolis supplementation on the severity of IBS symptoms.

## 2 MATERIALS AND METHODS

### 2.1 Study design and participants

This randomized, double‐blind, placebo‐controlled clinical trial was conducted on subjects with IBS diagnosed by a gastroenterologist according to the Rome IV criteria. Fifty‐six patients by simple random sampling were recruited from the Soroush Special Clinic of Ahvaz, Iran, between September 2019 and January 2020.

Based on the Rome IV criteria, patients who had recurrent abdominal pain on average at least 1 day/week in the last 3 months were identified as an IBS if he/she had at least two of the following criteria:

- Related to defecation.
- Associated with a change in frequency of stools.
- Associated with a change in form (appearance) of stools.

The inclusion criteria in this trial included patients aged 18–65 years who have a constipation subtype of IBS (IBS‐C) or a mixed subtype of IBS (IBS‐M) based on the Bristol stool form scale (BSFS); have no allergy to bee products; and had filled out a written consent form. The exclusion criteria of the study were pregnancy or breastfeeding; patients with malignancy or other chronic GI diseases; regular use of drugs that modify GI movements (such as metoclopramide, cisapride, narcotics, diphenoxylate, etc.); regular use of laxatives and/or antibiotics; the history of major surgery in the digestive system (such as Billroth's operation, having an ostomy, and any resection of any part of the digestive tract); being on diet; regular use of prebiotic and/or probiotic compounds; and use of psychotherapy drugs. Patients taking less than 80% of their supplements, unwilling to continue collaboration in the study, experiencing severe physical and mental trauma, or changing their diet plan or physical activity during the study were withdrawn from the trial.

The trial protocol, available at the Iranian Registry of Clinical Trials (https://en.irct.ir/trial/40983, registration number: IRCT20190708044154N1), was approved by the Ethics Committee of Tabriz University of Medical Sciences (Ethics code: IR.TBZMED.REC.1398.473). This trial was conducted under the Declaration of Helsinki. All patients were provided verbally with information on the objectives, benefits, and possible health risks of the trial at the time of enrollment and then provided written informed consent.

### 2.2 Randomization and intervention

Eligible patients were randomly allocated in a 1:1 ratio to receive propolis or placebo tablets. A random‐number table was used to generate randomization sequences with a block size of 4 and stratification according to IBS subtypes and gender. The propolis and placebo tablets were prepared in precisely the same color, size, odor, and packaging. Also, numbered drug containers were used to conceal random allocation. Except for the pharmacist, the patients and investigators were unaware of treatment assignments.

### 2.3 Supplementation

The supplements were prepared by the Mashhad School of Pharmacy, Mashhad University of Medical Sciences, Iran, under the supervision of a clinical pharmacist. Propolis tablets consist of 450 mg of propolis extract (containing 90 mg of polyphenols and 67 mg of flavonoids), whereas the placebo tablets contain microcrystalline cellulose (a powder that has no taste, calories, smell, or nutrients) and various edible colors. The tablets were administered before lunch and dinner for 6 weeks. The optimal dosage of propolis (900 mg/day) was extracted from animal studies and its method is completely described in the published protocol article of this study (Miryan et al., 2020). Due to the similar mechanism of propolis and prebiotics and probiotics for intestinal microflora, a period of 6 weeks is adequate to boost intestinal microflora and/or GI symptoms in patients based on former studies (Basturk et al., 2016; Han et al., 2017). One of the researchers was responsible for follow‐up patients by phone calls, weekly. She asked each patient to report any adverse effect they may have experienced during the study, and to fill out the supplement checklist in which the patients recorded the supplements consumed. In each visit, compliance was assessed by the supplement checklists and by counting the return of uneaten supplements.

### 2.4 Primary outcome

The main outcome of the trial was the percentage of patients with an improvement of at least one grade of IBS disease from baseline to the sixth week of intervention. To assess IBS severity, the IBS symptom severity scale (IBS‐SSS) was used. It was filled out by patients pre‐ and postintervention. The IBS‐SSS questionnaire included five clinically applicable items over 10 days such as: Ι) the abdominal pain intensity, ΙΙ) the frequency of abdominal pain, ΙΙΙ) the abdominal distension intensity, IV) dissatisfaction with bowel movements, and V) the potential impact of IBS on the patient's daily life. The mean score of each scale is a maximum of 100 and the questionnaire total score reaches a maximum of 500, eventually. Scores of <75, 75–175, 175–300, and ≥300 points displayed mild, moderate, and severe grades of the IBS disease, respectively (Francis et al., 1997).

### 2.5 Secondary outcomes

The secondary outcomes were changes in body mass index (BMI) and waist circumference (WC) from baseline to the end of the sixth week. Weight was measured to the nearest 0.1 kg using a calibrated scale (Seca 831, Hamburg, Germany) with patients wearing light clothes and no shoes. Height was measured to the nearest 0.1 cm using a stadiometer (Seca 206, Hamburg, Germany). Then, the BMI was calculated as weight (kg)/height^2^ (m). WC was measured as the smallest circumference between the costal and iliac crests using a nonstretchable measuring tape to the nearest 0.1 cm.

### 2.6 Confounding factors’ assessment

Dietary intake was measured using three‐day food records (two nonconsecutive weekdays and one weekend) before, middle, and after the trial. Then, the amount of dietary energy and nutrient intakes of patients was obtained with the use of Nutritionist IV software. The intensity of habitual physical activity in metabolic equivalent of task (MET) was measured using the validated international physical activity questionnaire‐short form (IPAQ‐SF) before and after the trial (Craig et al., 2003). We also measured the anxiety state of patients as a potential confounding factor with the use of the Beck anxiety inventory (BAI) before and after the trial. The BAI is a reliable and valid instrument measuring anxiety (Fydrich et al., 1992), which has a score from 0 to 63 points with the higher scores indicating higher anxiety.

### 2.7 Statistical analysis

Statistical analysis was done using IBM SPSS Statistics software, version 16 (SPSS Inc., and Chicago, IL, USA). The sample size was 28 patients in each group by assuming a between‐group difference of 25% points in the main outcome based on a two‐sided significance level of 5%, a power of 80%, and a withdrawal rate of 20% with the use of A’Hern's single‐stage phase II methodology (A'hern, R., 2001; Basturk et al., 2016). Data were presented as mean (*SD*) for numerical data, frequency (percentage) for categorical variables, and median (25th, 75th) for values with skewed distribution. For evaluating the differences between the two groups at baseline, independent samples *t*‐test or Mann–Whitney *U* test were used for values with normal and non‐normal distribution, respectively. Paired‐samples *t*‐test and Wilcoxon's signed‐rank test were used for assessing within‐group changes, as appropriate. For adjusting the confounding factors, the analysis of covariance (ANCOVA) test was used. The adjusted odds of improvement in IBS symptoms were calculated with the use of binary logistic regression. *p*‐values less than .05 were considered to indicate statistically significant differences.

## 3 RESULTS

**FIGURE 1:**
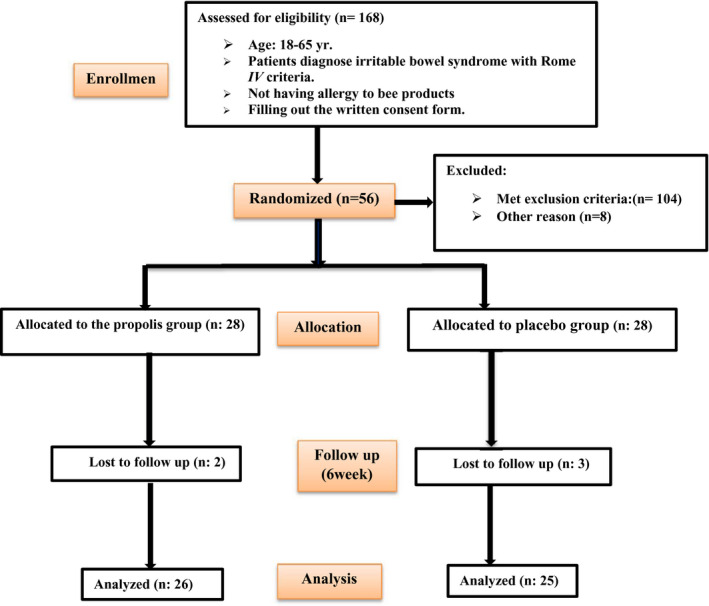
Study flow of enrollment, allocation, intervention, and assessment

A total of 168 patients were enrolled in the trial and were screened, of whom 56 patients met eligibility criteria and underwent randomization. Fifty‐one patients completed the trial, while three patients in the placebo group and two patients in the propolis group discontinued the trial for a reason unrelated to the trial. The trial flowchart is shown in Figure 1. There were no significant differences in terms of compliance rates between the propolis and placebo groups at the end of the trial (93% for propolis versus. 90% for placebo; *p*‐value = .73).

The demographic characteristics of the patients in both groups are shown in Table 1. At the baseline, there were no statistically significant differences between the two groups in terms of gender, marital status, education levels, occupational status, METs, IBS subtypes, disease duration, and anxiety state.

**TABLE 1:** Baseline characteristics of the study participants in both groups

Variables		Propolis group (*N* = 26)	Placebo group (*N* = 25)	*p*‐value
Age; years		38.92 ± 12.65	44.92 ± 12.10	.090^†^
Females; *n* (%)		13 (50%)	14 (56%)	.781^‡^
Married; *n* (%)		19 (73.07%)	23 (92%)	.076^‡^
Academic education; *n* (%)		10 (38.46%)	11 (44%)	.885^‡^
Employee; *n* (%)		20 (77%)	20 (80%)	.761^‡^
METs; minutes/week		691.50 [196.00–1629.00]	360.00 [173.25–1768.00]	.883*
Anxiety score		16.88 ± 11.01	17.68 ± 10.44	.793^†^
Disease duration; years		12.32 ± 10.5	8.31 ± 7.36	.366^†^
IBS Type	IBS‐C; *n* (%)	19 (73.07%)	17 (68%)	.764^‡^
	IBS‐M; *n* (%)	7 (26.92%)	8 (32%)	

Abbreviations: IBS, Irritable bowel syndrome; IBS‐C, Constipation subtype of IBS; IBS‐M, Mixed subtype of IBS; METs, Metabolic equivalents.

Physical activity levels are presented as median [25th, 75th]. Age and duration of IBS are presented as mean±*SD*; other variables are presented as number (%).

^†^ Values were obtained from independent samples *t*‐test.

^‡^ Values were obtained from the chi‐square test.

* Values were obtained from Wilcoxon rank‐sum test.

The adjusted mean changes in dietary intakes from baseline to the end of the trial in both groups are shown in Table 2. At baseline, the mean intakes of energy and nutrient had no significant differences between the propolis and placebo groups (*p*‐value > .05). The intakes of energy, macronutrients, lactose, and caffeine had no significant changes from baseline to the end of the trial in both groups (*p*‐value > .05). None of the participants in the present study reported alcohol consumption at the beginning and during the study. As shown in Table 3, there was no significant change in terms of weight, BMI, and WC from baseline to the end of the trial in both groups (*p*‐value > .05).

**TABLE 2:** Adjusted mean changes in dietary intakes from baseline to the end of the trial in both groups

Variables	Group	Before	After	*p*‐value^†^	Changes^‡^	*p*‐value^‡^
Energy; Kcal/day	Propolis	1495 ± 454	1559 ± 421	.604	−40.19 ± 18.22	.213
	Placebo	1712 ± 523	1486 ± 449	.099	−100.51 ± 19.86	
Protein; g/day	Propolis	60.96 ± 20.26	63.90 ± 21.55	.525	−2.74 ± 0.53	.553
	Placebo	74.49 ± 35.15	65.13 ± 22.71	.182	−5.03 ± 0.57	
Fat; g/day	Propolis	25.35 ± 10.02	29.79 ± 9.62	.104	−0.877 ± 0.511	.679
	Placebo	34.52 ± 14.21	30.69 ± 14.83	.481	0.671 ± 0.558	
Carbohydrate; g/day	Propolis	247.4 ± 99.7	255.1 ± 90.4	.771	−19.92 ± 2.04	.347
	Placebo	265.3 ± 89.5	237.2 ± 84.5	.171	−5.98 ± 2.22	
Dietary fiber; g/day	Propolis	12.18 ± 9.03	11.12 ± 4.21	.609	−2.25 ± 0.193	.195
	Placebo	12.30 ± 5.95	11.34 ± 5.70	.459	−0.42 ± 0.210	
Fructose; g/day	Propolis	6.39 ± 7.37	8.34 ± 7.01	.200	0.224 ± 0.219	.913
	Placebo	9.23 ± 8.31	8.69 ± 7.18	.685	0.399 ± 0.241	
Lactose; g/day	Propolis	4.09 ± 4.28	3.91 ± 3.56	.840	−0.369 ± 0.141	.748
	Placebo	4.24 ± 3.20	3.88 ± 3.06	.623	−0.040 ± 0.153	
Caffeine; mg/day	Propolis	334 ± 1221	416 ± 1.553	.811	−0.355 ± 0.120	.560
	Placebo	1341 ± 4655	1148 ± 3.938	.813	0.145 ± 0.125	

Abbreviation: IBS, Irritable bowel syndrome.

Data are presented as mean ±standard deviation.

^†^ Values were obtained from paired‐sample *t*‐test.

^‡^ Values were obtained from the analysis of covariance (ANCOVA) test with baseline values and changes in energy intake as covariates.

**TABLE 3:** Adjusted mean changes in anthropometric parameters in both groups

Variables	Group	Before	After	*p*‐value^†^	Changes^‡^	*p*‐value^‡^
Weight; kg	Propolis	72.10 ± 13.65	72.28 ± 13.84	.717	0.114 ± 0.094	.677
	Placebo	75.64 ± 15.08	75.61 ± 15.08	.939	0.063 ± 0.102	
BMI; kg/m^2^	Propolis	25.61 ± 4.00	25.58 ± 3.96	.711	−0.088 ± 0.028	.775
	Placebo	27.75 ± 5.85	27.73 ± 5.81	.877	0.023 ± 0.031	
WC; cm	Propolis	85.94 ± 15.77	87.28 ± 2.45	.711	1.34 ± 0.245	.593
	Placebo	95.20 ± 4.00	94.96 ± 2.74	.784	−0.23 ± 0.268	

Abbreviations: WC, Waist circumference; BMI, Body mass index.

Data are presented as mean ±standard deviation.

^†^ Values were obtained from paired‐sample *t*‐test.

^‡^ Values were obtained from the analysis of covariance (ANCOVA) test with baseline values and changes in physical activity and energy intake as covariates.

At the end of the trial, the median [interquartile range (IQR)] of METs was 662 [160–1840]and 691.5 [371.25–1400] minutes/week in the placebo and propolis groups, respectively. In both groups, there were no significant changes in METs from baseline to the end of the trial (*p*‐value > .05) (Table S1). At the end of the trial, the mean (standard deviation (*SD*)) of anxiety score was 16.72 (8.46) in the placebo group and 11.19 (8.37) in the propolis group. The anxiety score was significantly decreased in the propolis group compared with the placebo group (−5.69 ± 8.22 versus. −0.96 ± 7.81; *p*‐value = .40) (Table S2).

**FIGURE 2:**
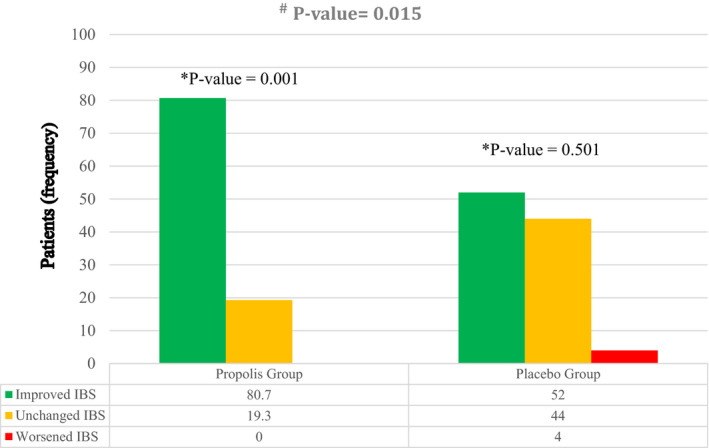
The changes in the degree of irritable bowel syndrome (IBS) from baseline to 6‐week intervention in the propolis and placebo groups. ^*^Within‐group comparisons with the use of a Wilcoxon rank‐sum test showed a significant improvement in the degree of IBS in the propolis group from baseline to 6‐week intervention. ^#^Mann–Whitney U test showed that the improvement in the degree of IBS in the propolis group was significantly higher than in the placebo group (*p*‐value = .015)

Adjusted mean changes in the primary outcome from baseline to the end of the trial are shown in Table 4. At baseline, overall scores of IBS symptoms and scores of all its components were similar in both groups (*p* > .05). Overall scores of IBS symptoms and scores of all their components were significantly reduced in the propolis group at the end of the trial (*p* < .05). In the placebo group, the severity of abdominal distension was significantly decreased, while other components did not change at the end of the trial (*p*‐value < .05). After the adjustment of anxiety score as a covariate, there were significant between‐group differences for the mean changes in overall scores of IBS symptoms, the severity of abdominal pain, and frequency of abdominal pain (*p*‐value < .05). In addition, the percentage of patients achieving at least one grade reduction in the IBS symptoms was significantly higher in the propolis group than in the placebo group (80.7% versus 52%; *p* = .015) (Figure 2). Patients in the propolis group were 6.22 times more likely to experience improvement in IBS symptoms than those in the placebo group (95% CI: 1.14–33.9; *p *= .035), independent of changes in anxiety scores.

**TABLE 4:** Adjusted mean changes in irritable bowel syndrome symptoms severity score (IBS‐SSS) in both groups throughout the trial

Variables	Group	Before	After	*p*‐value^†^	Changes^‡^	*p*‐value^‡^
Severity of abdominal pain	Propolis	55.76 ± 28.16	33.07 ± 26.94	.002	−24.75 ± 28.66	.004
	Placebo	55.60 ± 35.55	53.60 ± 29.98	.664	0.139 ± 28.66	
Frequency of abdominal pain	Propolis	5.15 ± 3.35	3.07 ± 3.22	.021	−2.24 ± 3.51	.041
	Placebo	5.08 ± 3.39	4.80 ± 2.76	.552	−0.11 ± 3.51	
Severity of abdominal distension	Propolis	64.61 ± 32.02	42.69 ± 25.22	.001	−22.77 ± 27.65	.328
	Placebo	63.60 ± 22.70	48.80 ± 19.64	.010	−14.44 ± 27.65	
Dissatisfaction with bowel habits	Propolis	60.00 ± 26.83	42.69 ± 23.24	.002	−16.89 ± 29.07	.060
	Placebo	64.40 ± 26.93	64.00 ± 24.15	.949	−0.83 ± 29.07	
Interference with quality of life	Propolis	46.92 ± 35.52	34.23 ± 28.16	.038	−11.97 ± 35.7	.372
	Placebo	55.60 ± 29.45	53.60 ± 24.81	.802	−2.75 ± 35.7	
Overall score	Propolis	288.84 ± 111.40	183.46 ± 106.46	.001	−98.27 ± 105.44	.011
	Placebo	290.00 ± 108.97	268.00 ± 81.54	.253	−18.99 ± 105.44	

Abbreviation: IBS, Irritable bowel syndrome.

Data are presented as mean ± standard deviation.

^†^ Values were obtained from paired‐sample *t*‐test.

^‡^ Values were obtained from the analysis of covariance (ANCOVA) test with the adjustment of changes in anxiety scores.

## 4 DISCUSSION

The main finding of this trial is that the administration of propolis (900 mg/day) for 6 weeks can improve the severity of IBS symptoms, which was mainly related to the severity and frequency of abdominal pain. Patients in the propolis group were 6.22 times more likely to experience improvement in IBS symptoms than those in the placebo group. To our knowledge, this is the first clinical trial providing evidence of the efficacy of propolis supplementation on IBS symptoms. Previous studies showed that IBS would be related to microscopic gut inflammation (Abboud et al., 2013). A recent systematic review concluded that the administration of propolis can be effective in improving many aspects of clinical, macroscopic, and histological features of inflammatory bowel disease in animal models (Soleimani et al., 2020). Propolis is a natural product containing a wide range of polyphenolic and flavonoid compounds that act in an antioxidant network. Our findings on the improvement of the IBS symptoms are in line with the results of previous clinical trials on dietary supplements. A clinical trial showed that the combination of green tea (containing epigallocatechin gallate, epigallocatechin, epicatechin gallate, and epicatechin polyphenols), curcumin, and selenomethionine for 4 weeks has a positive effect on satisfaction with bowel habits in patients with IBS (Lior et al., 2019). Another clinical trial indicated that the co‐supplementation of curcumin and fennel essential oil for 30 days improves IBS‐SSS, abdominal pain, and quality of life in IBS patients over 30 days (Portincasa et al., 2016). Quercetin glycosides represent the predominant flavonoid fraction in propolis. The administration of quercetin in postinflammatory IBS rats reduces the visceral motor response, 5‐hydroxytryptamine levels, and visceral motor response and increases the pain threshold pressure (Qin et al., 2019). Nonspecific propolis extract is involved in the immune response by activation of macrophages through releasing hydrogen peroxide and inhibiting the production of nitric oxide (dose‐dependent effect), which can be affected by inhibition of inducible nitric oxide synthase (iNOS) gene expression and iNOS catalytic activity (Orsi et al., 2000; Tan‐no et al., 2006). Some of the specific effects shown by the aqueous form of propolis include an inhibitory effect on platelet aggregation, an inhibitory effect on the synthesis of prostaglandins in vitro, and inhibition of 5‐lipoxygenase (5‐LOX) (Dobrowolski et al., 1991; Khayyal et al., 1993; Massaro et al., 2011). Studies have also shown that alcoholic propolis extract inhibits transcription of the iNOS gene through its effect on nuclear factor‐kappa B (NF‐κB) sites in the NF‐κB promoter, which is dose‐dependent (Song et al., 2002). Moreover, alcoholic extract of propolis can interfere with inflammatory response mechanisms, which have a very important effect on controlling cellular epithelial function (Xuan et al., 2010).

Our results also showed that the administration of propolis did not affect dietary intakes. This finding is consistent with those of previous studies in the context of energy and nutrient intakes. The results reported by Soleimani et al. showed that the administration of 900 mg/day of propolis for 4 months had no significant effect on energy and adjusted nutrient intakes in patients with nonalcoholic fatty liver disease (NAFLD) (Soleimani et al., 2021). Samadi et al. reported that the administration of 900 mg/day of propolis supplement for 3 months did not affect energy and nutrient intakes in patients with type 2 diabetes mellitus (T2DM) (Samadi et al., 2017). Similarly, Zhao et al. showed that the administration of 900 mg/day of propolis for 18 weeks did not affect energy and nutrient intakes in patients with T2DM (Zhao et al., 2016). Furthermore, another study revealed that the administration of a high dose of propolis (1500 mg/day) for 8 weeks had no significant effect on energy and nutrient intakes in patients with T2DM (Afsharpour et al., 2019). Therefore, it seems that the administration of propolis supplements in different doses and durations does not affect the amount of energy and nutrient intake.

Our results also showed that the administration of propolis did not affect anthropometric indices, including weight, BMI, and WC, independent of physical activity and energy intake. In line with this finding, Soleimani et al. reported no significant effect of propolis supplementation at a daily dose of 900 mg/day for 4 months on weight, fat mass, and fat‐free mass in patients with NAFLD after the adjustment of energy intake and physical activity as covariates (Soleimani et al., 2021). Likewise, Mujica et al. reported that the administration of propolis at a daily dose of 30 drops (3% propolis extract) for 3 months did not affect weight, BMI, and WC in subjects with cardiometabolic risk factors (Mujica et al., 2017). Also, Afsharpour et al. found that the administration of 1500 mg/day of propolis for 8 weeks did not affect body weight and BMI in patients with T2DM (Afsharpour et al., 2019). Nevertheless, the results reported by Samadi et al. showed a significant reduction in weight and BMI with the administration of 900 mg/day of propolis for 3 months in T2DM (Samadi et al., 2017). It seems likely that this inconsistency may be dependent on the effect of confounding factors such as changes in dietary intakes and physical activity levels throughout the study of Samadi et al.

The current trial had some strength. One of the most important strengths of this study was the good approach to the IBS patients with Rome IV criteria which is the best and newest tool for IBS diagnosis; also using stratified block randomization with a block size of 4 (based on IBS subtypes and gender) led to the distribution of features between the study groups. Another strong point of this study was the high compliance rate of patients for the treatment in each group. However, this trial had a few limitations including self‐reporting of physical activity and dietary intakes. The first limitation of this trial might be self‐reported physical activity and food intake. Another limitation was that there were no particular markers or laboratory parameters that are accessible to diagnose the disease. In this study, the best available diagnostic tool (which is a subjective questionnaire) was utilized for IBS diagnosis.

In conclusion, our results show that the administration of propolis may have a beneficial effect on the severity of IBS‐M and IBS‐C by reducing abdominal pain (severity and frequency), while it has no significant effect on dietary intakes and anthropometric indices in these subjects. Therefore, propolis can be used as adjunctive therapy in IBS‐M and IBS‐C to reduce abdominal pain. It is recommended that future studies specifically investigate the effects of propolis supplementation on diarrhea‐predominant IBS (IBS‐D) and gut microbiome of IBS patients.

## CONFLICT OF INTEREST

The authors have declared no conflict of interest.

## ETHICS STATEMENT

The trial protocol was approved by the Research Ethics Committee at the Tabriz University of Medical Sciences (IR.TBZMED.REC.1398.473). All patients provided written consent for participation in this study. In the study, we used the Rome IV questionnaires for IBS disease (the Rome IV diagnostic questionnaire for IBS disease, Persian versions of IBS‐SSS, and IBS Quality of Life Instrument (IBS‐QOL) questionnaires) after obtaining correspondence and authorization from Rome Foundation. The questionnaires were provided to the researchers under a contract.

## Supporting information

Table S1

Table S2

## Data Availability

The data that support the findings of this study are available on request from the corresponding author. The data are not publicly available due to privacy or ethical restrictions.
