# Supplementary material for: Effects of propolis supplementation on irritable bowel syndrome with constipation (IBS‐C) and mixed (IBS‐M) stool pattern: A randomized, double‐blind clinical trial
Source: Food Sci Nutr. 2022 Apr 20;10(6):1899–907. doi: 10.1002/fsn3.2806 (PMC9179135; doi:10.1002/fsn3.2806)
Supplement: Supplementary file 1 — Table S1 [file FSN3-10-1899-s001.docx]

| **Supplemental Table 1.** Physical activity values in the propolis and placebo groups before and after the trial. | | | |
| --- | --- | --- | --- |
| Physical activity | Placebo group  (N=25) | Propolis group  (26= N) | P-value ^b^ |
| Before; METs | 360 [173.25 - 1768] | 691.5 [196 - 1629] | 0.883 |
| After; METs | 662 [160 - 1840] | 691.5 [371.25 - 1400] | 0.472 |
| P-value ^a^ | 0.145 | 0.154 |  |
| **Abbreviation.** METs, Metabolic equivalents  Values ​​are presented as median [25th, 75th].  ^a^ P-values ​​were obtained from Wilcoxon rank-sum test.  ^b^ P-values ​​were obtained from Mann-Whitney U test. | | | |
