# Supplementary material for: Effects of propolis supplementation on irritable bowel syndrome with constipation (IBS‐C) and mixed (IBS‐M) stool pattern: A randomized, double‐blind clinical trial
Source: Food Sci Nutr. 2022 Apr 20;10(6):1899–907. doi: 10.1002/fsn3.2806 (PMC9179135; doi:10.1002/fsn3.2806)
Supplement: Supplementary file 2 — Table S2 [file FSN3-10-1899-s002.docx]

| **Supplemental Table 2.** Evaluation of patients' anxiety status in the propolis and placebo groups before and after the trial. | | | |
| --- | --- | --- | --- |
| Anxiety | Placebo group  (N=25) | Propolis group  (26= N) | P-value ^b^ |
| Before | 17.68 ± 10.44 | 16.88±11.01 | 0.793 |
| After | 16.44± 8.33 | 11.19±8.37 | 0.029 |
| Change | - 0.96 ± 7.81 | - 5.69± 8.22 | 0.040 |
| P-value ^a^ | 0.462 | 0.002 |  |
| Values ​​are presented as mean ± standard deviation.  ^a^ P-values ​​were obtained from Paired- sample t-test.  ^b^ P-values ​​were obtained from independent sample t-test. | | | |
